# Changes in the Prevalence of Diabetes Ketoacidosis at the Onset of Type 1 Diabetes in Polish Children: A Comparative Analysis Between Two 9-Year Periods—2006–2014 and 2015–2023

**DOI:** 10.1155/pedi/8927409

**Published:** 2025-07-23

**Authors:** Elżbieta Niechciał, Michał Bielecki, Adrianna Geppert, Sebastian Kokociński, Kamil Kopa, Patrycja Wiącek, Oliwia Witkowska, Laura Dwulit, Olga Mejer, Andrzej Kędzia

**Affiliations:** Department of Pediatric Diabetes, Clinical Auxology and Obesity, Poznan University of Medical Sciences, 27/33 Szpitalna Street, Poznan 60–572, Poland

## Abstract

**Objective:** Having been facing a progressive increase in the prevalence of type 1 diabetes (T1D), there might be a growing risk of the development of diabetic ketoacidosis (DKA) at disease onset. The prevalence of DKA varies widely by geographic region, ranging from approximately 13% in Sweden to 80% in the United Arab Emirates. This study aimed to compare the prevalence of DKA in childhood-onset T1D from Greater Poland (Poland) in two 9-year periods.

**Methods:** We assessed the prevalence of DKA in children aged <18 years with newly diagnosed T1D in Greater Poland (Poland) in two 9-year periods, 2006–2014 and 2015–2023, in a retrospective review of a complete regional cohort. DKA and its severity were classified according to International Society for Pediatric and Adolescent Diabetes (ISPAD) guidelines.

**Results:** Over the 18 years, 2432 children below 18 years of age with newly diagnosed T1D were recorded. The overall prevalence of DKA was 51.3% (*n* = 1248), and it rose significantly in two nine-year periods, from 47.7% in 2006–2014 to 53.4% in 2015–2023 (*p*=0.007). There was a significant shift toward more severe presentations of DKA. While the prevalence of mild DKA decreased slightly from 51.3% to 47.0% (*p*=0.145), and moderate DKA had a notable decline from 33.1% to 25.2% (*p*=0.003), the proportion of severe DKA cases rose sharply from 15.5% to 27.7% (*p* < 0.001).

**Conclusions:** Despite the increasing incidence of T1D in Poland, healthcare, and parental awareness of T1D symptoms remain low, which results in delayed T1D recognition. The escalating prevalence of DKA at T1D onset in children is a concerning public health issue that necessitates a multifaceted approach to education, prevention, and early intervention. Addressing these challenges might help reduce the prevalence of DKA and improve clinical outcomes for children with T1D.

## 1. Introduction

The current epidemiological data indicate a constant increase in the prevalence of childhood type 1 diabetes (T1D) worldwide. It is estimated that there are around 1.2 million children and adolescents under 20 years who had T1D in 2021, with 150,000 new cases diagnosed that year [1]. An annual incidence increase ranges from 3% to 5% globally, with younger individuals experiencing the most significant rise [2, 3]. The incidence rate of T1D varies widely by geographic region and ethnic population. European countries still have the highest number of newly diagnosed cases of T1D in children and adolescents, with the most rapid rate of increase contributed by countries in Central and Eastern Europe, including Poland, which also experienced a sharp growth in childhood T1D incidence [1, 4–8]. Caucasian people seem to be more susceptible to T1D than other ethnic groups. However, rising trends are also observed in several populations outside of Europe [1].

Having been facing a progressive increase in the prevalence of T1D, there might be a growing risk of the development of acute and chronic associated complications, including diabetic ketoacidosis (DKA). However, there is conflicting data on the relationship between the increase in the incidence of T1D and its influence on DKA prevalence at diagnosis. Some longitudinal studies reported a reduction in DKA prevalence with a parallel rise in T1D incidence [9, 10], while others did not confirm this observation [11–13]. Nevertheless, the higher background of T1D incidence is considered a protective factor against DKA development at disease onset [14].

The prevalence of newly diagnosed T1D presenting as DKA varies widely by geographic region, ranging from approximately 13% in Sweden to 80% in the United Arab Emirates. The main risk factors associated with DKA occurrence at T1D presentation include young age (mainly <2 years), minority ethnicity, poor access to medical care, diagnostic error, delayed treatment, lack of health insurance, lower socioeconomic status, preceding infection, while, factors such as first degree relative with T1D or higher parental education are considered as a protective factor.

DKA is a life-threatening complication defined as a biochemical triad of hyperglycemia, ketonemia, and acidemia with sudden symptom onset. There is a global variation in mortality related to DKA at the first T1D presentation in children and adolescents. In developed countries, the overall mortality is estimated at 0.15% to 0.35%, while in developing countries, clinic-based studies found rates ranging from 3.4% to 13.4%. Cerebral edema, sepsis, and renal failure are the most important triggering factors of DKA-associated mortality in the developing world [15]. Moreover, late presentation in DKA at T1D onset is not only responsible for a more significant risk of morbidity and mortality, but also is the main issue from the economic point of view because it also increases health care costs. Most children in DKA might require admission to the intensive care unit, which extends the duration of hospitalization and might cause various complications that drive up costs [16, 17]. Finally, recent studies showed that the development of DKA at T1D onset is related to a poorer prognosis and less residual β-cell function after diagnosis and, as an independent factor, negatively influences long-term metabolic control of T1D [18, 19]. This study aimed to compare the prevalence of DKA in childhood-onset T1D from Greater Poland (Poland) in two 9-year periods, 2006–2014 and 2015–2023.

## 2. Methods

This retrospective cohort study collected data for children and adolescents living in Greater Poland (west-central Poland) with newly diagnosed T1D admitted to the Department of Pediatric Diabetes, Clinical Auxology and Obesity in Poznan (Poznan University of Medical Sciences). Hospital inpatient clinic medical records were reviewed and met the following criteria: New-onset T1D (confirmed by the presence of diabetes-associated autoantibodies) diagnosed between 1 January 2006 and 31 December 2023, age 0–18 years, permanent residency at the time of diagnosis in the study area, which was defined geographically to correspond with administrative and census boundaries of Greater Poland. As a retrospective medical chart review was performed, it was exempt from the Bioethical Committee review. Still, we respected the principles of the Declaration of Helsinki and protected patients' confidentiality.

The following data were collected from hospital inpatient medical records: Age, sex, and laboratory tests at diagnosis (blood glucose, pH, HCO3, HbA1c, and C-peptide). Diabetes was diagnosed in accordance with the International Society for Pediatric and Adolescent Diabetes (ISPAD) criteria. The first day of insulin administration was at T1D diagnosis. We applied the current biochemical criteria for the diagnosis of DKA recommended by the ISPAD: hyperglycemia (blood glucose concentration >200 mg/dL [>11 mmol/L]), blood pH <7.30, the concentration of bicarbonate HCO3 <18 mmol/L, in addition to ketonemia and ketonuria. Metabolic acidosis was considered mild, moderate, and severe if pH and bicarbonates were <7.3 and 18 mmol/L, <7.2 and 10 mmol/L, and <7.1 and 5 mmol/L, respectively. According to the results, children were divided into the non-DKA and DKA groups. While based on age at the time of diagnosis, participants were classified into five age subgroups: 0–2 years old, 3–6 years old, 7–10 years old, 11–14 years old, and 15–18 years old. Finally, the change in DKA prevalence in the analyzed population between 2006–2014 and 2015–2023 was investigated.

Fasting C-peptide was assessed to measure residual β-cell function at T1D onset. C-peptide concentration was analyzed using radioimmunoassay (C-PEP II-RIA-CT, DIAsource Immunoassay, S.A, Louvain-la-Neuve, Belgium), with the normal range: 0.59–1.54 pmol/mL. Glycated hemoglobin (HbA1c) was measured using the NGSP-certified method (HbA1c ARCHITECT System, Abbott Laboratories, Illinois, USA).

### 2.1. Statistical Analysis

This study considered two-sided *p*-values less than α = 0.05 statistically significant. Descriptive statistics were employed to summarize the data. The normality of numerical variables was assessed using the Shapiro–Wilk test. For continuous variables that deviated from normality, the median (Mdn) was selected as the measure of central tendency due to its robustness to outliers, and the first (Q1) and third (Q3) quartiles were reported to represent the interquartile range (IQR). Categorical variables were summarized using frequency (n) and percentage (%). The Wilcoxon rank-sum test was applied to compare differences between two independent groups for continuous variables that did not follow a normal distribution. Group comparisons were conducted using Pearson's chi-square test for categorical variables.

The 95% confidence intervals (CIs) for DKA prevalence was calculated using the Wilson Score Interval method. To assess temporal changes in DKA prevalence between the two time periods, the difference in proportions was calculated, and 95% CIs for the difference were estimated using the normal approximation method. The standard error for the difference in proportions was computed as the square root of the sum of the variances of the individual proportions, with the CI constructed as the difference ± 1.96 times the standard error. Analyses were conducted using the R Statistical language (version 4.3.3; R Core Team, 2024).

## 3. Results

Over the 18-year period from 2006 to 2023, a total of 2432 children below 18 years of age with newly diagnosed T1D from Greater Poland were recorded. The prevalence of T1D diagnosis was more common in males, and it was reported that 54.9% of patients were male (*n* = 1335) and 45.1% female (*n* = 1097). The median age at diagnosis was 9.0 years (Q1:5.0, Q3:12.0). DKA was diagnosed in 51.3% (*n* = 1248) of patients with newly diagnosed T1D, of whom 24.9% (*n* = 606) developed mild, 14.3% (*n* = 349) moderate, and 12.0% (*n* = 293) severe. Characteristics of the study population at the time of T1D diagnosis are presented in Table 1.

A notable correlation was found between sex and the prevalence of DKA at diagnosis, with a greater percentage of females diagnosed with DKA (47.2%) than those without it (42.9%; *p*=0.034). Additionally, the median age for patients presenting with DKA was significantly younger (9.0 years; Q1:4.0, Q3:12.0) compared to those without DKA (9.0 years; Q1:6.0, Q3:13.0), with a statistically significant difference (*p*  < 0.001). Furthermore, blood glucose and HbA1c levels were found to be considerably higher in children experiencing DKA (*p*  < 0.001), whereas C-peptide levels were notably lower (*p*  < 0.001). A comparison of demographic and clinical features and characteristics in all patients with and without DKA at T1D onset is summarized in Table 2.

When comparing the prevalence of DKA in childhood-onset T1D from Greater Poland in two 9-year periods, 2006–2014 and 2015–2023, a significant increase in the number of children presenting DKA was observed over the two time periods (*p*=0.007). Between 2006 and 2014, 47.7% of newly diagnosed T1D cases presented with DKA, whereas from 2015 to 2023, this figure rose to 53.4%.  Table 3 shows the change in the DKA prevalence between the two analyzed periods. While Table 4 examines the temporal changes in the severity of DKA at the time of T1D onset, stratified by age group, comparing two periods: 2006–2014 and 2015–2023. The table highlights shifts in the prevalence of mild, moderate, and severe DKA, providing insights into trends over time, both for the pediatric population and specific age groups.

For all age groups combined, there was a significant shift toward more severe presentations of DKA. While the prevalence of mild DKA decreased slightly from 51.4% to 47.1% (*p*=0.145), and moderate DKA saw a notable decline from 33.1% to 25.2% (*p*=0.003), the proportion of severe DKA cases rose sharply from 15.5% to 27.7% (*p* < 0.001). The most pronounced changes were observed in the youngest children (0–2 years) when stratified by age group. In this group, the prevalence of severe DKA increased significantly from 16.7% to 39.1% (*p*=0.013), while the prevalence of mild DKA dropped from 50.0% to 32.7% (*p*=0.062), though the latter did not reach statistical significance. A similar trend was observed in the 7–10 year age group, with severe DKA increasing from 14.4% to 29.2% (*p*=0.003) and moderate DKA decreasing significantly from 38.1% to 24.8% (*p*=0.012). In contrast, the 11–14 year age group also saw a significant increase in the prevalence of severe DKA, rising from 13.2% to 27.4% (*p*=0.002), while mild DKA decreased from 56.2% to 46.9% (*p*=0.094). In the 15–18 year age group, the prevalence of severe DKA increased from 13.3% in 2006–2014 to 23.4% in 2015–2023, a change of + 10.11% (95% CI: −5.2, 25.4; *p*=0.188), which was not statistically significant. This increase is comparable in magnitude to the significant increase observed in the 11–14 year age group (+ 14.17%, 95% CI: 4.8, 23.5; *p*=0.002), though the smaller sample size in the 15–18 year group (*n* = 21 vs., *n* = 82) results in greater uncertainty, as evidenced by the wide CI. The lack of statistical significance in the 15–18 year group may reflect limited statistical power due to the small number of cases rather than a meaningfully smaller increase.

**Figure 1:**
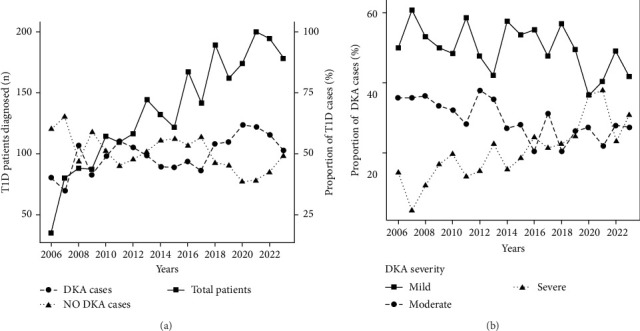
Analysis of temporal trends in type 1 diabetes diagnosis and diabetic ketoacidosis severity among pediatric patients from Greater Poland between 2006 and 2023. (a) A number of pediatric patients diagnosed with T1D (solid line with squared points) scales on the left *Y*-axis, the respective rate of DKA (dashed with round points), and non-DKA (dotted line with tingled points) scales on the right *Y*-axis. (b) The proportion of patients with mild (solid line with squared points), moderate (dashed with round points), and severe (dotted line with tingled points) DKA at T1D diagnosis. DKA, diabetic ketoacidosis.

Finally, Figure 1 illustrates the temporal trends in the number of pediatric patients diagnosed with T1D and the corresponding prevalence of DKA and non-DKA presentations at diagnosis. The prevalence of DKA at diagnosis fluctuated throughout the study period, with notable increases in recent years. In earlier years, such as 2006, the DKA prevalence was 40%, but this rate rose steadily, peaking at 61.4% in 2020. Similarly, high DKA rates were observed in 2021 (61.0%) and 2022 (57.7%). Moreover, there were notable shifts in the distribution of DKA severity during the study period. Early in the study period (e.g., 2006–2010), mild DKA was the predominant presentation, ranging from 48.2% to 60.7% of DKA cases. However, the proportion of severe DKA has increased significantly in recent years.

## 4. Discussion

In this study, we compared the prevalence of DKA at childhood-onset T1D from Greater Poland in two 9-year periods. Our analyses demonstrated a high overall prevalence of DKA at the first-time presentation of T1D, with a significant increase in its prevalence from 47.7% in 2006–2014 to 53.4% in 2015–2023. In Poland, a nationwide registry of DKA has not yet been established, but we have data from several diabetes centers in the country. This study's results align with data from other parts of our country, also showing a comparable prevalence of DKA among Polish children at the time of diagnosis [20]. Compared to other European countries, Poland has an unacceptably high prevalence of DKA in the childhood population [21]. This high trend cannot be explained by the low prevalence of T1D in children, as for over 20 years, Poland has been experiencing a systematic increase in the number of children with newly diagnosed T1D [6, 8]. The rise in DKA prevalence, in this study, during 2020 and 2021 may be partly attributed to the COVID-19 pandemic, which likely delayed healthcare access and early diagnosis, leading to more severe presentations of T1D [20].

Most research on DKA prevalence in children indicates that age is one of the most crucial risk factors for the development of DKA in the childhood population. Younger individuals have a greater risk of acidosis crisis at T1D onset. Similarly, in our study, the risk of DKA development decreased with age. In detail, children under the age of two had a high prevalence of DKA, exceeding 76.0%, while adolescents were characterized by the lowest DKA prevalence, estimated at 41.6%. Several reasons are responsible for the increased prevalence of DKA in younger children. One of the causes might be lower parental and/or health provider awareness, which can lead to a slow recognition or misinterpretation of diabetes symptoms. In consequence, it delays diabetes diagnosis and treatment. Previous data from Greater Poland strongly suggest that diabetes misdiagnosis, symptoms duration over 28 days, and age under 4 are the most significant factors negatively affecting the DKA rate. However, younger children might present more aggressive autoimmune processes than older individuals. Then, the pancreatic β-cell function can decline faster, and DKA occurs more rapidly in such young individuals [22].

DKA is a life-threatening condition with possible high mortality and well-documented negative impact on long-term disease management. Our study not only showed a rise in DKA prevalence, but more importantly, an increase in the number of cases presenting with severe DKA at the time of childhood T1D diagnosis. This substantial increase in severe DKA prevalence over time emphasizes a concerning trend, suggesting that a larger proportion of children are being diagnosed at a more critical stage of metabolic decompensation. This may indicate delays in diagnosis and treatment, potentially due to factors such as late symptom recognition or reduced access to timely medical care. Even in older children, where symptom recognition might be expected to be more straightforward, in this study, the delay in diagnosis persists, resulting in more severe DKA at presentation. Therefore, some initiatives aiming to reduce DKA prevalence at the disease onset seem to be reasonable options for early T1D recognition. Some countries have already implemented public educational campaigns, achieving excellent results by reducing the prevalence of DKA at disease onset. Recently, Cherubini et al. delivered evidence of the effectiveness of social campaigns in increasing awareness about DKA in children by conducting a systematic review and meta-analysis. The analysis included 14 studies focused on community-based interventions in children below 18 years, and the authors measured only the prevalence of DKA as an outcome. In this systematic review, the pooled difference was a reduction of 7.2% (95% CI: 0.99–13.41) [23]. However, educational campaigns should not only be addressed to society but also to healthcare professionals, especially pediatricians, primary care clinicians, and nurses. Greater awareness and skills in identifying T1D symptoms might help reduce the prevalence of DKA at the first presentation of the disease. An example of such a program was a multi-intervention project implemented in a Canadian province. The aim of this project was to reduce the prevalence of DKA in children and young adults and was directed to the groups mentioned above. During the 6-year period of the project, a decrease in the hospitalization rate due to DKA was observed in pediatric patients in the province. Interestingly, the hospitalization rate after the project period returned to a value similar to that applicable before the project, which shows the effectiveness of the campaign, although, at the same time, routine actions are necessary to maintain a constant reduction in hospitalization rate due to DKA [24]. Another study was performed in the UK, and it also emphasized the significance of detecting and targeting factors related to DKA development at T1D onset. In this study, parents of newly diagnosed children and medical teams involved in the diagnostic process were asked to complete a questionnaire about events before diagnosis. The reported results showed that a greater parental awareness of typical T1D symptoms (polyuria and/or polydipsia) was associated with timely T1D diagnosis and DKA reduction. Moreover, this study demonstrated that health professional education targeting nonclassical symptoms, particularly among those below 2 years of age, and point-of-care testing benefited in decreasing the prevalence of DKA at diagnosis [25]. Similar effects have been observed in other countries, such as France, Turkey, and Germany, where campaigns aimed at health professionals and parents helped to improve the early recognition of T1D [26]. One of the most extensive programs was the Parma campaign conducted in Italy. The project involved educational intervention for doctors, teachers, and parents. Over the 8 years of this initiative, a significant reduction in DKA prevalence at T1D diagnosis was observed; the cumulative prevalence of DKA decreased from 78% to 12.5%, and, more importantly, its long-term effects remained several years after it was promoted [27, 28].

Another strategy that may help reduce DKA, which has been increasingly emphasized recently, is multiple islet autoantibodies screening in individuals at high risk and in the general population. The data from many countries, such as the U.S., Germany, Finland, Sweden, Australia, and New Zealand, have demonstrated that children with detected multiple islet autoantibodies who were monitored had low DKA rates of ≤6% [29–33]. Screening programs focusing on detecting islet autoantibodies can provide benefits not only for the early detection of T1D but also for offering participants with positive autoantibodies some prevention intervention. Currently, such programs based on primary or secondary prevention are mainly offered in clinical trials. However, this seems to be the future of diabetic care for children with T1D. For example, TrialNet is an international research program aimed at preventing the development of T1D and developing new treatment methods [34]. This program addresses relatives of people with T1D, people at high risk of developing the disease (with some genetic markers), and in the early stages of the disease. Screening tests are based on checking for the presence of disease-related antibodies. If the participant is eligible, they might be included in the prevention study called the anti-thymocyte globulin (ATG) Prevention Study (STOP-T1D, Study ID NCT04291703). This study tests a low dose of the immunotherapy drug ATG to examine if it can delay or even prevent T1D [35]. So far, ATG has been approved by the Food and Drug Administration (FDA) only for preventing and treating kidney transplant rejection. However, it might be officially introduced to T1D treatment if it shows positive results. One example of a treatment already approved by the FDA is teplizumab, an anti-CD3 antibody that can delay the development of T1D for several years [36]. In Poland, several programs also aim to promote the early recognition of T1D in high-risk populations. For instance, there is the SINT1A project, among others [37]. The study is part of an international initiative run under the Global Platform for the Prevention of Autoimmune Diabetes (GPPAD). The aim is to screen for antibodies and monitor children with genetic predispositions to detect T1D earlier. However, information about such programs is mainly disseminated in large diabetes centers. So far, they are primarily targeted at children having first-degree relatives with T1D rather than the general population. Therefore, educational programs addressing the symptoms of T1D and point-of-care testing still seem to be the most optimal strategy for reducing DKA at the onset of childhood T1D. However, a significant drawback of these programs is the need for frequent repetition to maintain awareness of the disease. This can generate substantial financial costs; however, it still appears that treating DKA requires even greater financial resources.

The findings presented in our study are crucial for understanding evolving patterns in disease presentation, particularly the increasing prevalence of severe DKA in recent years. The ability to identify which age groups are most affected by these changes can inform targeted interventions aimed at early diagnosis and prevention of severe metabolic complications in children with newly diagnosed T1D.

## 5. Strengths and Limitations

The strength of the present study is a relatively long-term period of observation, which allows for understanding trends in the prevalence of DKA at childhood onset of T1D in Greater Poland. In addition, Greater Poland is second in area and third in population among Poland's 16 voivodeships, so it might be representative of the Polish population. Nevertheless, some limitations of the current study need to be acknowledged. Pediatric diabetes care is centralized in Greater Poland, and almost all children newly diagnosed with diabetes from the study area are referred to the Department of Pediatric Diabetes, Clinical Auxology and Obesity in Poznan (Poznan University of Medical Sciences). However, this geographical selection may introduce selection bias, and then we cannot be assured that we have collected all cases. Some cases of newly diagnosed T1D could have been referred to a center in another voivodeship, especially if these individuals lived in border areas.

## 6. Conclusions

Despite the increasing incidence of T1D in Poland, healthcare and parental awareness of T1D symptoms remain low, which results in delayed T1D recognition. Consequently, during 18 years of study, the number of cases presenting DKA at T1D onset exceeded more than 50%. There was a significant increase in DKA prevalence at T1D diagnosis in children below 18 years of age from Greater Poland. DKA prevalence rose from 47.7% in 2006–2014 to 53.4% in 2015–2023. For all age groups combined, there was also a significant shift toward more severe presentations of DKA. While the prevalence of mild DKA decreased slightly from 51.3% to 47.0%, and moderate DKA saw a notable decline from 33.1% to 25.2%, the proportion of severe DKA cases rose sharply from 15.5% to 27.7%. The escalating prevalence of DKA at T1D onset in children is a concerning public health issue that necessitates a multifaceted approach to education, prevention, and early intervention or screening programs. Addressing these challenges might help reduce the prevalence of DKA and improve clinical outcomes for children with T1D.

## Figures and Tables

**Table 1:** Baseline characteristics of the study participants at T1D diagnosis (2006–2023).

Characteristic	*N*	Distribution^a^
Sex	2432	—
Female	—	1097 (45.1%)
Male	—	1335 (54.9%)
Age (years)	2432	9.0 (5.0, 12.0)
Age group	2432	—
0–2 years	—	191 (7.9%)
3–6 years	—	565 (23.2%)
7–10 years	—	650 (26.7%)
11–14 years	—	764 (31.4%)
15–18 years	—	262 (10.8%)
Glycemia (mg/dL)	2432	379 (292, 498)
pH	2431	7.36^b^ (7.25, 7.40)
HCO_3_ (mmol/L)	2431	17.90^b^ (9.60, 21.80)
NGSP HbA1c (%)	2431	11.2^b^ (9.7, 12.9)
C-peptide level (pmol/L)	2432	0.29^b^ (0.21, 0.40)
DKA	2432	1248 (51.3%)
Severity of the of DKA	2432	—
Mild	—	606 (24.9%)
Moderate	—	349 (14.3%)
Severe	—	293 (12.0%)

*Note*: HbA1c, glycated hemoglobin.

Abbreviations: DKA, diabetic ketoacidosis; T1D, type 1 diabetes.

^a^ *n* (%).

^b^Mdn (Q1, Q3).

**Table 2:** Comparison of demographic and clinical features in patients with and without DKA at T1D diagnosis.

Characteristic	DKA		*p*
	Yes, *n*_1_ *=* 1248^a^	No, *n*_2_ *=* 1184^a^	
Sex (F:M), *n* (%)	589 (47.2%):659 (52.8%)	508 (42.9%):676 (57.1%)	0.034
Age (years)	9.0^b^ (4.0, 12.0)	9.0^b^ (6.0, 13.0)	<0.001
Glycemia (mg/dL)	409^b^ (321, 522)	348^b^ (271, 467)	<0.001
NGSP HbA1c (%)	11.7^b^ (10.2, 13.1)	10.7^b^ (9.0, 12.6)	<0.001
C-peptide level (pmol/L)	0.26^b^ (0.19, 0.33)	0.36^b^ (0.25, 0.47)	<0.001

*Note*: HbA1c, glycated hemoglobin.

Abbreviation: DKA, diabetic ketoacidosis.

^a^ *n* (%).

^b^Mdn (Q1, Q3).

**Table 3:** The increasing prevalence of DKA at T1D onset in children from Greater Poland in two 9-year periods: 2006–2014 and 2015–2023.

Characteristic	Total (*n*)	2006–2014 (*n*_1_ *=* 905)	2015–2023 (*n*_2_ *=* 1,527)	*p*
DKA prevalence, *n* (% [95% CI])	2432	432 (47.7% [44.5–50.9])	816 (53.4% [50.9–55.9])	0.007

Abbreviations: CI, confidence interval; DKA, diabetic ketoacidosis; T1D, type 1 diabetes.

**Table 4:** Temporal changes in DKA prevalence at T1D diagnosis in children from Greater Poland in two 9-year periods: 2006–2014 and 2015–2023, stratified by DKA severity and age group.

Age group (years)	DKA severity	Total (*n*)	2006–2014 *n* (%) [95% CI]	2015–2023 *n* (%) [95% CI]	Change (%)	95% CI for change (%)	*p*
All age groups (*n* = 1248)	Mild	606	222 (51.4%) [46.6–56.0]	384 (47.1%) [43.6–50.4]	−4.33	[−10.1, 1.4]	0.145
	Moderate	349	143 (33.1%) [28.7–37.7]	206 (25.2%) [22.3–28.3]	−7.85	[−13.3, −2.3]	0.003
	Severe	293	67 (15.5%) [12.3–19.3]	226 (27.7%) [24.6–30.9]	+12.18	[7.0, 17.3]	<0.001

0–2 (*n* = 146)	Mild	54	18 (50.0%) [34.1–65.8)	36 (32.7%) [24.4–42.2]	−17.27	[−35.0, 0.5]	0.062
	Moderate	43	12 (33.3%) [20.0–49.9]	31 (28.2%) [20.4–37.4]	−5.15	[−22.3, 12.0]	0.556
	Severe	49	6 (16.7%) [7.6–32.4]	43 (39.1%) [30.3–48.6]	+22.42	[7.0, 37.7]	0.013

3–6 (*n* = 304)	Mild	157	54 (48.2%) [39.1–57.4]	103 (53.6%) [46.4–60.7]	+5.44	[−6.5, 17.4]	0.361
	Moderate	84	36 (32.1%) [24.2–41.2]	48 (25.0%) [19.2–31.8]	−7.14	[−18.3, 4.1]	0.179
	Severe	63	22 (19.7%) [13.2–28.2]	41 (21.4%) [16.0–27.8]	+1.71	[−9.0, 12.4]	0.723

7–10 (*n* = 327)	Mild	152	56 (47.5%) [38.4–56.6]	96 (45.9%) [39.1–52.8]	−1.53	[−13.6, 10.5]	0.791
	Moderate	97	45 (38.1%) [29.6–47.3]	52 (24.9%) [19.3–31.3]	−13.26	[−24.4, −2.0]	0.012
	Severe	78	17 (14.4%) [9.1–22.0]	61 (29.2%) [23.3–35.7]	+14.78	[4.8, 24.6]	0.003

11–14 (*n* = 362)	Mild	181	68 (56.2%) [47.0–64.9]	113 (46.9%) [40.5–53.3]	−9.31	[−20.8, 2.2]	0.094
	Moderate	99	37 (30.6%) [22.9–39.3]	62 (25.7%) [20.4–31.7]	−4.85	[−15.3, 5.6]	0.329
	Severe	82	16 (13.2%) [8.2–20.6]	66 (27.4%) [22.0–33.4]	+14.17	[4.8, 23.5]	0.002

15–18 (*n* = 109)	Mild	62	26 (57.8%) [42.9–71.2]	36 (56.3%) [43.9–67.8]	−1.53	[−20.4, 17.4]	0.874
	Moderate	26	13 (28.9%) [17.6–43.5]	13 (20.3%) [12.3–31.6]	−8.58	[−24.8, 7.6]	0.301
	Severe	21	6 (13.3%) [6.1–26.9]	15 (23.4%) [14.6–35.2]	+10.11	[−5.2, 25.4]	0.188

Abbreviations: CI, confidence interval; DKA, diabetic ketoacidosis; T1D, type 1 diabetes.

## Data Availability

The datasets used in this study are available from the corresponding author upon reasonable request.
